# Long-term self-reported symptoms and psychophysical tests in COVID-19 subjects experiencing persistent olfactory dysfunction: a 4-year follow-up study

**DOI:** 10.3389/fncir.2025.1538821

**Published:** 2025-05-07

**Authors:** Tommaso Saccardo, Giuseppe Roccuzzo, Alessandro Fontana, Sonny Zampollo, Bruno Scarpa, Piero Nicolai, Alfonso Luca Pendolino, Carla Mucignat, Rosario Marchese-Ragona, Giancarlo Ottaviano

**Affiliations:** ^1^Otolaryngology Section, Department of Neurosciences, University of Padova, Padova, Italy; ^2^Department of Statistical Sciences, University of Padova, Padova, Italy; ^3^Department of Mathematics “Tullio Levi-Civita”, University of Padova, Padova, Italy; ^4^Ear Institute, University College London, London, United Kingdom; ^5^Department of Molecular Medicine, University of Padova, Padova, Italy

**Keywords:** smell loss, parosmia, Sniffin’ stick test, COVID-19, olfactory recovery

## Abstract

**Background:**

Since the onset of the COVID-19 pandemic, chemosensory dysfunction (CD), including olfactory and taste quantitative dysfunction (OD/TD), has emerged as a prevalent and early symptom in SARS-CoV-2-infected subjects. This study explores the prevalence, duration, and recovery trajectory of COVID-19-related olfactory dysfunction (C19OD), with a specific focus on the four-year follow-up.

**Methods:**

Using a combination of psychophysical tests (Sniffin’ sticks) and patient-reported outcome measures (sVAS and tVAS), 83 participants were prospectively evaluated for OD and parosmia. Factors influencing long-term olfactory recovery were analysed.

**Results:**

Baseline assessments revealed OD in 56.6% of patients, with progressive improvement observed over 4 years. At the four-year follow-up, 92.3% of patients recovered their olfaction while the remaining still reported hyposmia. Younger age and olfactory training were found to be favourable prognostic factors.

**Conclusion:**

Our findings show that, despite most individuals with C19OD recover olfaction within the first year, a subset of them continue to experience prolonged CD, demonstrating a slow, constant and meaningful improvement over years. This prolonged recovery period highlights the complexity of SARS-CoV-2’s impact on olfactory function and highlights the need of further research on CD pathophysiology with the aim to improve therapeutic approaches to C19OD.

## Introduction

Since the outbreak of the coronavirus disease 2019 (COVID-19) pandemic, the severe acute respiratory syndrome coronavirus-2 (SARS-CoV-2) has become one of the major causes of olfactory and taste quantitative dysfunction (OD, TD) in the population, together known as chemosensory dysfunction (CD). CD soon appeared to be a frequent and characteristic early symptom in a relevant percentage of SARS-CoV-2-infected patients (Giacomelli et al., 2020; Hornuss et al., 2020; Doty, 2022; Andrews et al., 2020) showing high variability in terms of severity, ranging from mild to complete loss of smell and taste (hyposmia/anosmia and hypogeusia/ageusia, respectively), and duration, lasting from only a few days to several months. Although the majority of COVID-19 patients experience symptoms resolution within 1 year, up to 28% of them can still report OD after 2 years and up to 5% after 3 years following SARS-CoV-2 infection (Boscolo-Rizzo et al., 2023a). As a result, CD represents one of the main features of long-COVID, significantly affecting patients’ quality of life (QoL) (Vaira et al., 2022; Saniasiaya and Prepageran, 2021; Pendolino et al., 2023b) and associated with increased level of depression and anxiety (Croy et al., 2014; Neuland et al., 2011).

Still today, the pathophysiology of COVID-19 CD and the factors influencing its highly-variable recovery have not been fully clarified. Older age has been identified as a negative prognostic factor for persistent COVID-19-related OD (C19OD) (Prem et al., 2024) while the role of parosmia is conflicting (Prem et al., 2024; Menzel et al., 2023). Moreover, prevalence of OD and recovery rate after 4 years still remain unknown. Considering the widespread of COVID-19 and the long-term impact of CD on patient’s QoL, it is important to acknowledge the long-term persistence of OD.

In a previous prospective study, we evaluated early characteristics of C19OD specifically looking at the correlation between patient-reported outcome measures (PROMs) and psychophysical tests, suggesting a potential end-organ failure pathogenesis of C19OD (Bordin et al., 2021). Given the unreliability of solely patient-reported olfactory function (Whitcroft and Hummel, 2020; Marchese-Ragona et al., 2020), we followed up our previous cohort of patients over a period of 4 years using both PROMs [visual analogue scale for smell (sVAS) and taste (tVAS)] and psychophysical tests (Sniffin’ sticks—S’S) with the aim to evaluate the long-term recovery trajectory of C19OD.

## Materials and methods

This study was conducted in accordance with the 1996 Helsinki Declaration (Hospital Research Ethics Committee Prot. 056881). Since March 2020, when OD and TD were identified as key symptoms of SARS-CoV-2 infection (Ottaviano et al., 2020), we have started recruiting COVID-19 patients complaining of CD. Patients were invited to complete a questionnaire on OD/TD along with a sVAS and a tVAS (0 corresponded to the worst thinkable situation and 10 to not affected) (Rimmer et al., 2019). Inclusion criteria were age >18 years old, a laboratory confirmation of SARS-CoV-2 infection [by reverse transcription polymerase chain reaction (RT-PCR)] as well as the recovery confirmation from the infection by three negative diagnostic nasal/throat swabs prior to the olfactory examination. Exclusion criteria were past history of OD and/or TD, head and neck tumours, previous chemotherapy or radiotherapy to the head and neck region, previous head trauma, history of chronic rhinosinusitis or neurological diseases. Informed consent was obtained from each participant before starting any study-related procedure. All the patients who completed the initial screening were then invited to undergo formal smell evaluation at the ear, nose and throat clinic of the same hospital (T0). Olfactory function was assessed by means of S’S, as previously described (Ottaviano et al., 2018; Whitcroft et al., 2023; Ottaviano et al., 2013a; Ottaviano et al., 2013b). According to the final S’S score (TDI) obtained by summing the threshold (T), discrimination (D) and identification (I) subtest scores, olfactory function was classified as functional anosmia (TDI ≤16), hyposmia (TDI between 16 and 30.75) and, normosmia (TDI ≥30.75) (Oleszkiewicz et al., 2019; Prem et al., 2022). Contextually, s/tVAS were administered and, in addition, presence of parosmia was investigated by asking the patients if they were experiencing a “distorted sense of smell.” Patients found to have an OD at T0 S’S were invited to start olfactory training (OT) (Hummel et al., 2017) which involved twice-daily exposure to four distinct odorants (rose, eucalyptus, lemon, and clove) for a period of at least 6 months, and to repeat olfactory evaluation 1 year after the presumed infection date (T1). Four years after SARS-CoV-2 infection, patients who were initially found to have an altered sense of smell at T1 were invited to have a second olfactory evaluation (T2). Furthermore, we also asked the subjects that presented OD at T0 and did not complete the first-year evaluation, to undergo a second test 4 years after the baseline evaluation.

### Statistical analysis

Paired Wilcoxon test was adopted to measure the difference in time of TDI and its components (T, D, and I), s/tVAS and the effect of sex and smoke. Bravais–Pearson correlation coefficient has been used to measure the relations between TDI scores an sVAS scores. Multivariate analysis was conducted to evaluate factors associated with improvement in TDI scores. A multiple linear regression model was applied, using a hybrid backward stepwise variable selection based on Akaike’s information criterion (AIC) to optimize model fit. The following variables were included in the analysis: age at baseline, sex, presence of allergies, presence of chronic rhinosinusitis, smoking habit, type of onset of olfactory loss (sudden vs. progressive), type of onset of hypogeusia (sudden vs. progressive), hypogeusia recovery status (no recovery, partial recovery, complete recovery), presence of parosmia, vitamin B1 or B12 supplementation, olfactory rehabilitation and time since first diagnosis.

For all tests, *p*-values were calculated, and 5% was considered as the critical level of significance. The R: a language and environment for statistical computing (R Foundation for Statistical Computing, Vienna, Austria) statistical package was used for all analyses [R “stats” (version 4.4.2), “MASS” (version 7.3–61), and “olsrr” (version 0.6.1)].

## Results

**Figure 1:**
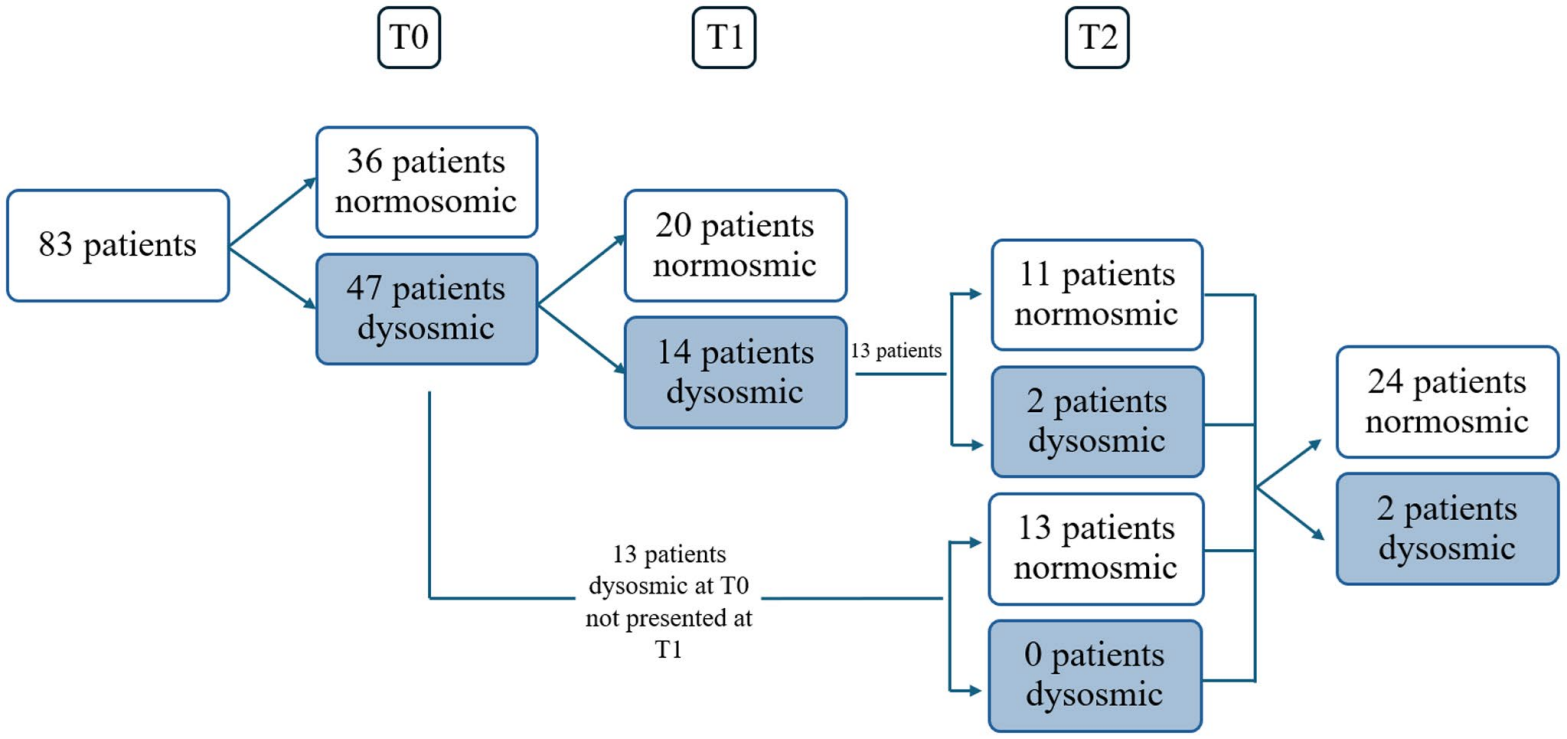
Schedule of visits and patients. Dysosmic: hyposmic-anosmic patients.

**Figure 2:**
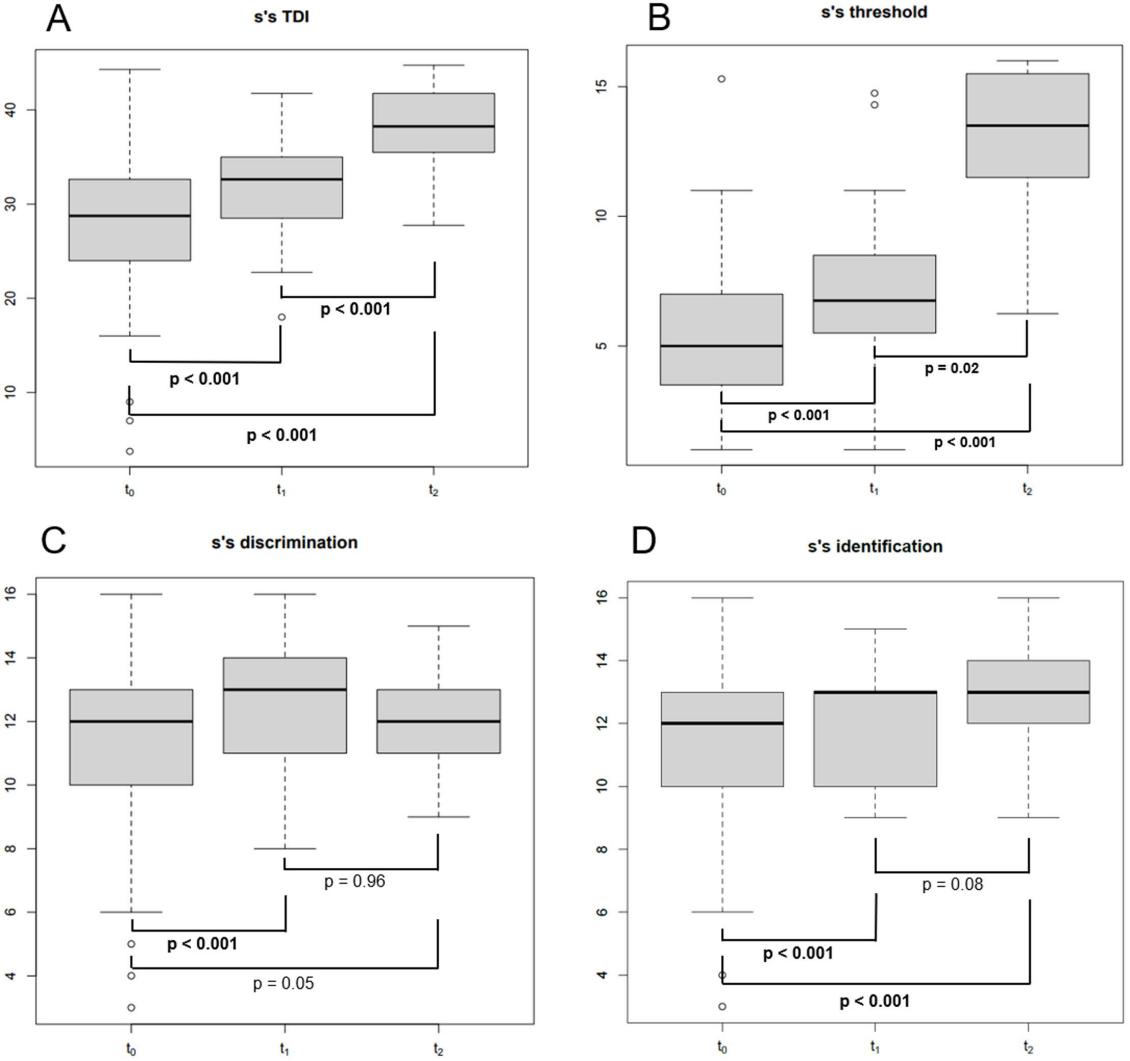
Boxplot showing Sniffin’ sticks total score (TDI) **(A)**, threshold score **(B)**, discrimination score **(C)** and identification score **(D)** at different follow-up times. S’S, Sniffin’ sticks; TDI, the total Sniffin’ sticks score; T0: baseline (mean of 2 months after SARS-CoV-2 infection); T1: 1 year after the infection (mean of 13 months after T0); T2: 4 years after the infection (mean of 35 months after T1); bold: statistically significant values. *p*-values were obtained for paired tests.

**Figure 3:**
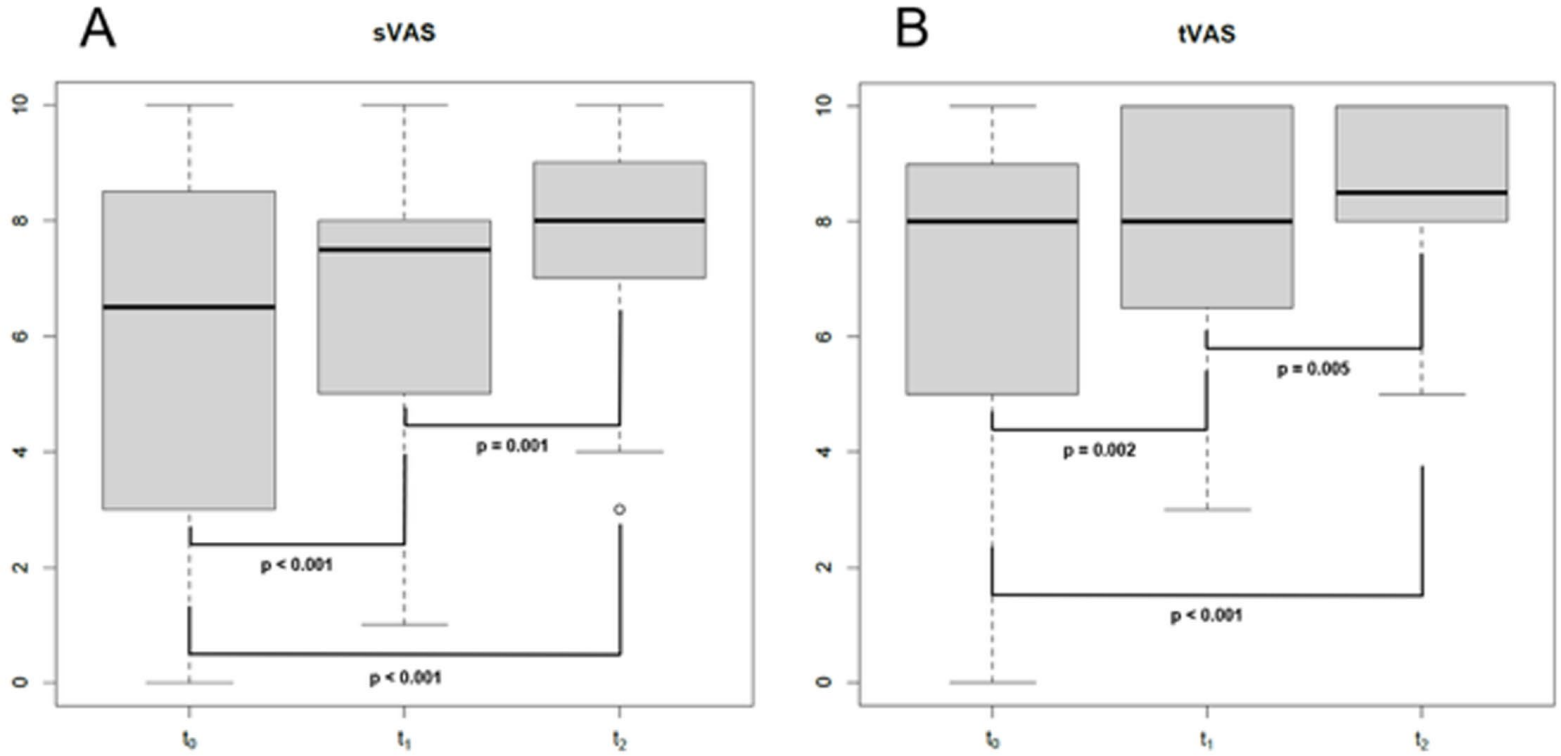
Boxplot showing sVAS **(A)** and tVASat different follow-up times. s/t, smell/taste; VAS, visual analogue scale; T0: baseline (mean of 2 months after SARS-CoV-2 infection); T1: 1 year after the infection (mean of 13 months after T0); T2: 4 years after the infection (mean of 35 months after T1); bold: statistically significant values. *p*-values were obtained for paired tests.

Eighty-three consecutive COVID-19 subjects (31 males and 52 females, mean age ± SD = 44.1 ± 15.5 years, 9 smokers and 2 with diabetes) (Table 1) complaining of CD underwent smell performance evaluation by means of S’S at T0. The mean follow-up time at T0 was 2.4 ± 2.2 months after diagnosis, being influenced by patients’ availability and the time needed to isolate and obtain a negative COVID-19 test. At T0, 36 patients were normosmic (36/83, 43.4%), 45 were hyposmics and 2 were anosmics (prevalence of OD was 56.6%, 47/83) (Figure 1) (Bordin et al., 2021). Amongst the 9 smokers evaluated at T0, 5 were normosmic, 3 hyposmic and 1 anosmic (Table 1). The mean TDI was 27.9 ± 7, mean T was 5.5 ± 2.9, mean D was 11.3 ± 2.9 and mean I was 11.7 ± 2.9 (Figure 2). sVAS was 6.4 ± 3.05 and tVAS was 7.4 ± 2.63 (Figure 3). Parosmia was reported by 12 patients (12/83, 14.5%) with 6 of them found to be normosmic and 6 hyposmic.

**Table 1:** Main demographics characteristics at follow-up visits.

	T0	T1	T2
Age	44.1 + 15.5	42.9 ± 14.2	47.1 ± 15.4
Sex	31 M 52 F	9 M 25 F	10 M 16 F
Smokers	9/83 (10.8%)	4/34 (11.7%)	3/26 (11.5%)
Hyposmic smokers	3/9 (33.3%)	3/4 (75%)	0
Anosmic smokers	1/9 (11.1%)	0	0
OT	N/A	26/34 (76.5%)	3/26 (11.5%)
Hyposmic on OT	N/A	11/26 (42.3%)	0
Smokers on OT	N/A	3/4 (75%)	0
Hyposmic smokers on OT	N/A	2/3 (66.7%)	0
B-Vitamins	N/A	25/34 (73.5%)	9/26 (34.6%)
Hyposmic on B-vitamins	N/A	10/34 (29.4%)	2/9 (22.2%)
Smokers on B-vitamins	N/A	2/4 (50%)	1/4 (25%)
Hyposmic smokers on B-vitamins	N/A	2/4 (50%)	0

OT, olfactory training; T0: baseline (mean of 2 months after SARS-CoV-2 infection); T1: 1 year after the infection (mean of 13 months after T0); T2: 4 years after the infection (mean of 35 months after T1); M, male; F, female; N/A, not applicable; TDI, total Sniffin’ sticks score; anosmia: TDI ≤16; hyposmia: TDI between 16 and 30.75.

Of the 47 patients with a confirmed OD at T0, 34 attended the second olfactory performance evaluation approximately 1 year after their SARS-CoV-2 infection onset (T1) (mean time from the infection: 15 ± 5.2 months; attendance rate: 72.3%). TDI scores showed persistent OD (hyposmia) in 14 patients (14/34, 41.2%), while 20 subjects were normosmic (20/34, 58.8%) (Figure 1). Among the 4 hypo-anosmic smokers at T0 who came for T1 evaluation, the anosmic one at T0 became hyposmic, 2 remained hyposmic and only 1 became normosmic (Table 1). Looking at the TDI score and subscores, mean TDI was 31.6 ± 5, mean T was 7.1 ± 2.9, mean D was 12.5 ± 1.8 and mean I was 12 ± 1.8, confirming a statistically significant improvement for TDI (*p* < 0.001) and for all the subscores [T, D, and I (*p* < 0.001)] at T1 when compared to T0 (Figure 2). At T1, sVAS was 6.7 ± 2.5 and tVAS 7.7 ± 2.3 (Figure 3) demonstrating a statistically significant improvement for both (*p* < 0.001 and *p* = 0.002, respectively). Nine patients performed OT for at least 6 months during the first year, while 7 subjects also used oral vitamin B complex. Parosmia was reported by 18 patients (18/34, 52.9%), with 15 of them being hyposmic at S’S. Nineteen patients (19/26, 73%) reported at least one new COVID-19 infection during the follow-up before T2 timepoint.

**Figure 4:**
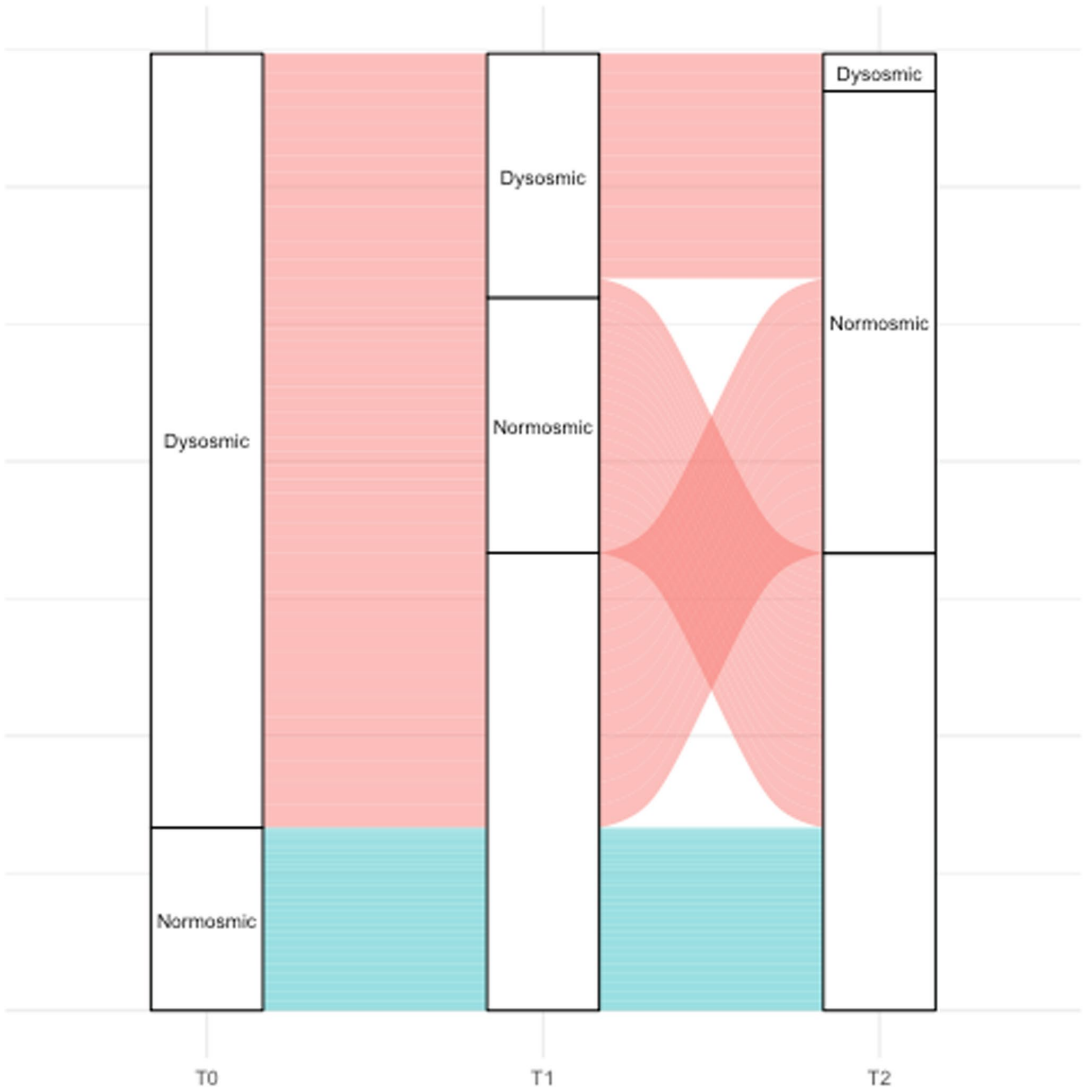
Alluvial plot showing patients’ recovery and retention throughout the study. Dysosmic: hyposmic-anosmic patients according to TDI. TDI, total Sniffin’ sticks score; dysosmic: TDI <30.75; normosmic: TDI ≥30.75.

At T2, only 13 of the patients found to have OD at T1 agreed to undergo a third olfactory evaluation (mean time from the infection: 49.8 ± 4.4 months) (attendance rate: 13/14, 92.9%). Only two of these patients were still hyposmic (15.4%) (Figure 1) Among the hyposmic smokers at T1, none was hypo-anosmic at T2 (Table 1). When attempting to re-evaluate the subjects found with OD at T0, but who did not attend the T1 evaluation, 13 more patients accepted to undergo the 4-years olfactory evaluation (mean 50.1 ± 3.5 months from the infection). As none of these patients were found to be hypo-anosmic, a total of 2 patients (2/26, 7.7%) were hyposmic at T2, while all the others were normosmic (24/26, 92.3%) (Figure 1). The mean TDI score for the 26 patients was 38 ± 4.4, the mean T score was 12.8 ± 2.9, the mean D was 12.1 ± 1.7, the mean I was 13.6 ± 1.8. TDI showed a statistically significant improvement at T2 with respect to T1 (*p* < 0.001). Considering the subscores T, D, and I, we found a statistically significant improvement for T (*p* = 0.002), but not for D and I (*p* = 0.96, *p* = 0.08, respectively) (Figure 2). Considering s/tVAS, at T2 the former was 7.6 ± 1.8, while the latter was 8 ± 1.4. Both s/tVAS showed a statistically significant improvement at T2 with respect to T1 (*p* = 0.001 and *p* = 0.005, respectively) and to T0 (*p* < 0.001 for both) (Figure 3). The main olfactory results are reported in Table 2. An alluvial plot describes patient recovery and retention in the study (Figure 4).

**Table 2:** Main olfactory results at the different follow-up visits.

Parameter	T0 (83 patients)	T1 (34 patients)	T2 (26 patients)	p-value (T1 vs. T0)	p-value (T2 vs. T1)	p-value (T2 vs. T0)
TDI score, mean ± SD	27.9 ± 7	31.6 ± 5	38 ± 4.4	***p* < 0.001**	***p* < 0.001**	***p* < 0.001**
Threshold (T), mean ± SD	5.5 ± 2.9	7.1 ± 2.9	12.8 ± 2.9	***p* < 0.001**	***p* = 0.002**	***p* < 0.001**
Discrimination (D), mean ± SD	11.3 ± 2.9	12.5 ± 1.8	12.1 ± 1.7	***p* < 0.001**	*p* = 0.96	*p* = 0.06
Identification (I), mean ± SD	11.7 ± 2.9	12 ± 1.8	13.6 ± 1.8	***p* < 0.001**	*p* = 0.08	***p* < 0.001**
sVAS, mean ± SD	6.4 ± 3.05	6.7 ± 2.5	7.6 ± 1.8	***p* < 0.001**	***p* = 0.004**	
tVAS, mean ± SD	7.4 ± 2.63	7.7 ± 2.3	8 ± 1.4	***p* < 0.001**	***p* = 0.003**	
Normosmic, n (%)	36/83 (43.4)	20/34 (58.8)	24/26 (92.3)	—	—	
Hyposmic, n (%)	45/83 (54.2)	14/34 (41.2)	2/26 (7.8)	—	—	
Anosmic, n (%)	2/83 (2.4)	0/34	0/26	—	—	
Parosmic, n (%)	12/83 (14.5)	18/34 (52.9)	6/26 (23.1)	—	—	

VAS, visual analogue scale; s, smell; t, taste; T0: baseline (mean of 2 months after SARS-CoV-2 infection); T1: 1 year after the infection (mean of 13 months after T0); T2: 4 years after the infection (mean of 35 months after T1); TDI, total Sniffin’ sticks score; SD, standard deviation; *n*, number of patients; hyposmia: TDI between 16 and 30.75; normosmia: TDI ≥30.75. Bold: statistically significant values.

With regards to olfactory therapy between T1 and T2, 9 patients continued to take oral B vitamins complex for a minimum of 2 months, while 6 patients performed OT for longer than 6 months. At T2 parosmia was reported only by 6 patients (23.1%), with 1 of them being disosmic at S’S.

Considering the correlation between the subjective perception of olfaction in terms of sVAS and the S’S scores, it was found a significant positive correlation only at T0 (*r* = 0.49, *p* < 0.001) and T1 (*r* = 0.41, *p* < 0.001). When looking at the potential role of sex, no significant differences in TDI scores between sexes were observed at T0, T1, or T2. Additionally, no differences by sex emerged in TDI scores between T1 and T0 and between T2 and T0 both at univariate and multivariate analyses.

The multiple multivariate analyses showed a statistically significant positive effect of age (younger), presence of olfactory training, and smoking on TDI score improvement (Table 3).

**Table 3:** Multivariate regression model to predict which variables may influence olfactory recovery.

	Estimate	Std. error	*t* value	*p*-value
(Intercept)	18.96960	1.74611	10.864	1.79 × 10^−6^
Age	−0.25122	0.04264	−5.891	0.000232
Smoke	9.50790	1.74266	5.456	0.000403
Olfactory training	5.86315	1.48693	3.943	0.003390

## Discussion

The long-term impact of COVID-19 on CD continues to be a relevant area of research, particularly given the persistence of OD in some patients years after infection. Current literature supports the notion that while many individuals recover their sense of smell within 2 weeks (Santos et al., 2021), a subset of patients experience prolonged OD (Liao et al., 2023). Studies have reported that between 5 to 10% of individuals who experience C19OD may not fully recover their sense of smell after 2 years and report long-standing CD impairment (Boscolo-Rizzo et al., 2024b). Moreover, previous research showed that the majority of patients with long-standing CD are still from the first wave of the pandemic which may be explained by the different SARS-CoV-2 variants (mainly alpha variant during the first wave) selected over the last 4 years with potential different pathogenicity on the olfactory epithelium. Moreover, an influence of COVID-19 vaccination on variants’ selection must be taken into account. The majority of our patients have been infected in the early months of the year 2020 by the first alpha variants of SARS-CoV-2. Mutations in D614G, which enhance viral penetration into host cells, likely accounted for the variation in prevalence of CD seen in the early phases of the pandemic (von Bartheld et al., 2021). With the appearance of other SARS-CoV-2 variants, the prevalence of taste and smell dysfunction has markedly decreased. More recent studies (Vihta et al., 2022; Vaira et al., 2023; Boscolo-Rizzo et al., 2023b) reported a prevalence of smell and taste dysfunction of 13–18% in patients affected by the Omicron variant, compared with 44–72% of patients with the previous variants.

In the present study the OD prevalence at baseline was 56.6% (Bordin et al., 2021). The analysis of the S’S scores at T0 showed that odour threshold was the most affected olfactory ability (reference values according to Oleszkiewicz et al., 2019). Moreover, while at T1 all the three S’S sub-scores increased significantly, at T2 only odour threshold demonstrated a significant improvement. Our data seem to suggest that odour threshold is not only the most impaired olfactory ability but also the last one to recover, as already noted in previous publications (Favero et al., 2022), or the one more likely to remain affected long-term. Since both odour discrimination and identification are thought to be functionally related to higher brain functions, whereas the odour threshold reflects the function of the peripheral olfactory system, our study suggests that persistent C19OD is mainly caused by a damage/impairment of the olfactory epithelium.

PROMs (sVAS and tVAS) showed a constant and statistically significant improvement over time, confirming our previous findings (Pendolino et al., 2023a). This subjective improvement was confirmed by a parallel improvement in the psychophysical tests. In fact, a complete recovery of olfactory function was achieved in 92.3% of our patients. As expected, the improvement in olfactory function was accompanied by a parallel subjective enhancement in gustatory function. Our results corroborate results reported by other authors when looking at C19OD recovery after 3 years (Boscolo-Rizzo et al., 2024a).

One of the most controversial findings in the recent literature is the heterogeneity in C19OD recovery rate. While some individuals experience full recovery, others report only a partial improvement or a persistent severe dysfunction (Dias et al., 2024). Although sex differences were not significant in the incidence of anosmia or in COVID-19 recovery rates (Pierron et al., 2020), some studies indicate that female subjects are more likely to report persistent OD, although this may reflect a higher baseline olfactory sensitivity in women (Oliveira-Pinto et al., 2014). Notably, a pre-COVID meta-analysis demonstrated that women outperform men in most aspects of olfaction (Sorokowski et al., 2019) and it is possible that a stronger baseline olfactory ability could cause females to be more sensitive to detect smell changes, and as a result they would be more prone to report subjective olfactory changes (Dias et al., 2024). In our cohort, the number of women complaining of OD at baseline was approximately 1.7 times higher than men. This difference decreased over time, and at the last follow-up, the prevalence of persistent OD was the same in both sexes. Nevertheless, according to our analyses, sex did not play a significant role in the severity of olfactory dysfunction or in the recovery process.

In our cohort, the number of patients complaining of parosmia at baseline was 12/83 (14.5%) with 50% of them being hypo-anosmics. One year later (T1), the number of patients complaining of parosmia was 18/34 (52.9%) with 7 of them being hypo-anosmics, while at T2 only 6 of them (23.1%) still complained of persisting parosmia with none of them being hyposmic at TDI test. The influence of parosmia on olfactory recovery is controversial, and over the years studies have shown this to be either a positive or a negative prognostic factor (Menzel et al., 2023; Prem et al., 2024). Onset of parosmia following a COVID-19 infection has been extensively reported, often as a delayed presentation either weeks or months after the initial infection (Duyan et al., 2021). While parosmia has been associated with olfactory neuron regeneration in post-infectious olfactory dysfunction (Liu et al., 2021), indicating some level of sensory recovery, on the other side it has been shown to reflect a depletion of the correctly functioning neuronal pool leading to an incorrect characterisation of odours (Dias et al., 2024). As a result, patients experiencing parosmia often report a poorer QoL and a worse orthonasal olfactory function (Menzel et al., 2023; Pendolino et al., 2023a). In our cohort, however, parosmia was not correlated with OD recovery. Furthermore, the fact that most patients with persistent parosmia displayed good orthonasal function suggests that some of the mechanisms involved in quantitative olfactory recovery may be involved in qualitative olfactory changes as well.

The correlation between self-rated olfactory alteration (sVAS) and the psychophysical olfactory tests at T0 and T1 was significant, but moderate (*r* = 0.49, *p* < 0.001 at T0 and *r* = 0.41, *p* < 0.001 at T1) and not significant at T2 suggesting that COVID-19 patients may estimate incorrectly their sense of smell, especially in the long-term. This further highlights the importance of measuring the sense of smell in these patients adopting psychophysical olfactory tests, such S’S.

Our multivariate analyses also showed younger age to be an independent favourable prognostic factor of olfactory recovery. It is possible that younger people, due to their better neural plasticity (Huart et al., 2019), may display a faster healing process of the olfactory epithelium after infection (Butowt et al., 2023). Our paper shows that the rate of complete olfactory recovery after 4 years from initial infection is above 90%, suggesting that in few patients the regenerative processes of olfactory neurons may take longer.

Olfactory training and smoke also emerged as independent favourable prognostic factors. Olfactory training has extensively proven to be effective in both the acute and chronic phases of post viral OD (Hwang et al., 2023; Boscolo-Rizzo et al., 2024a). In literature, several other therapeutic approaches have been investigated but their true efficacy remains unproven (O’Byrne et al., 2022; Hu et al., 2023; Lechien et al., 2024). In our cohort, surprisingly, smoking was found to be an independent positive prognostic factor for OD recovery. However, this is in contrast with recent results from a large American study on long-term taste and smell function recovery where smoking did not reach any statistical significance (Sharetts et al., 2024). Even though our finding could have been influenced by a small sample bias, with a higher representation of smokers in our cohort, it corroborates previous results from other authors who found a positive influence of smoking on both discrimination and identification scores, as well as a lower prevalence of C19OD in patients with a smoking habit (Pendolino et al., 2023a; Akbari et al., 2022; Thakur et al., 2022; Printza et al., 2021).

Potential limitations of the present study include: (I) the single-centre design, (II) the relatively small number of participants (*n* = 83), (III) the heterogeneity of treatments tried by patients for OD, (IV) the lack of assessment of olfactory function before SARS-CoV-2 infection, since all longitudinal studies need to account for the background prevalence of hyposmia in the general population of nearly 20% (Brä et al., 2004), (V) the lack of patients’ SARS-CoV-2 vaccination data due to the fact that all of them were infected and reported OD before vaccines became available, (VI) the fact that only hypo-anosmic patients were included at the subsequent follow-up visits after T0. Nevertheless, after 4 years only one patient of those found hypo-anosmic at T0 was lost at 4-year follow-up.

## Conclusion

In conclusion, this article contributes to the growing evidence that olfactory recovery after SARS-CoV-2 infection can still happen even after 4 years. Given the complex and variable nature of C19OD, further studies are needed to understand the virus’s pathophysiology and the factors influencing persistent post-viral hypo/anosmia and parosmia. Both quantitative and qualitative olfactory dysfunction remain an important public health problem with notable implications on patients’ QoL and mental wellbeing.

## Data Availability

The raw data supporting the conclusions of this article will be made available by the authors, without undue reservation.
